# Altered DNA methylation in neonates born large-for-gestational-age is associated with cardiometabolic risk in children

**DOI:** 10.18632/oncotarget.13442

**Published:** 2016-11-18

**Authors:** Xian-hua Lin, Dan-dan Wu, Ling Gao, Jun-yu Zhang, Hai-tao Pan, Hui Wang, Cheng Li, Ping Zhang, Meng-xi Guo, Yan-ting Wu, Ya-jing Tan, Li Jin, Yu-qian Xiang, Ju-xue Li, Jian-zhong Sheng, He-feng Huang

**Affiliations:** ^1^ The International Peace Maternity and Child Health Hospital, School of Medicine, Shanghai Jiao Tong University, Shanghai, China; ^2^ Institute of Embryo-Fetal Original Adult Disease, Affiliated to Shanghai Jiao Tong University, School of Medicine, Shanghai, China; ^3^ The Key Laboratory of Reproductive Genetics, Ministry of Education, Zhejiang University, Hangzhou, China; ^4^ Department of Pathology and Pathophysiology, School of Medicine, Zhejiang University, Hangzhou, China; ^5^ Department of Obstetrics and Gynecology, Meihua Central Hospital, Shanghai, China

**Keywords:** neonate, large-for-gestational-age, DNA methylation, preschool children, cardiometabolic risk

## Abstract

**Background:**

Infants being born Large-for-gestational-age (LGA) are prone to developing cardiometabolic disease. However, the underlying mechanisms remain unclear.

**Results:**

Clinical investigation showed that children born LGA had significantly higher serum level of total cholesterol (TC), low-density lipoprotein-cholesterol (LDL-c), and insulin, ratio of TC/high-density lipoprotein-cholesterol (HDL-c) compared to children born appropriate for gestational age (AGA). Birth weight (BW) was positively correlated to TC, LDL-c, and the ratio of TC/HDL in serum. Genome-wide DNA methylation analyzed in umbilical cord blood of controls and macrosomia cases. We identified 3459 methylation variable positions (MVPs) achieving genome-wide significance (adjusted *P*-value < 0.05) with methylation differences of ≥ 5%. A total of 327 MVPs were filtered by methylation differences of ≥ 7% located within an island, which mapped to 213 genes. Function analysis using Ingenuity Pathway Analysis showed 16 genes enriched in “cardiovascular disease”. Four genes included contributed to hyperlipidemia.

**Materials And Methods:**

Fifty-eight children aged 3–6 years born LGA and 123 subjects born AGA were enrolled. Anthropometric parameters and blood pressure (BP) were measured, and metabolic assessment was performed in all subjects. Genome-wide DNA methylation in umbilical blood was assayed by the 450K BeadChip in six AGA and six macrosomia newborns.

**Conclusions:**

Our data indicate that excess birth weight may increase the risk of lipid dysfunction in children aged 3–6 years. It might through reprogramming a group of genes correlated to cardiovascular disease. The genes identified in this study might be potential biomarker for cardiometabolic disease.

## INTRODUCTION

Prenatal and early postnatal origins of cardiometabolic disease have been extensive studied in the past a few years. The gestational stage and early childhood represents a window of phenotypic plasticity and is a sensitive period related to programming cardiometabolic risk. Women with increased body mass index (BMI) and obesity during pregnancy commonly result in fetal overgrowth and the birth of a large-for-gestational age (LGA) infant [1]. Infants being born LGA are prone to developing obesity, diabetes, and hypertension during childhood and later in life [2, 3]. Being born LGA to mothers with or without gestational diabetes mellitus (GDM) and obesity is associated with diverse effects on cardiometabolic risk factors at prepuberty [4–6]. Other studies have shown that high birth weight and rapid weight gain in childhood are associated with cardiometabolic risk later in life [7–10].

Cumulative evidences suggest that there is a relationship between excess birth weight and metabolic syndrome (MetS) and cardiovascular disease (CVD) risk factors [4, 6]. However, the underlying mechanisms have not yet been clearly demonstrated. MetS and CVD have a heritable component that is not attributable to genetic factors. Instead, epigenetic mechanisms may have an additional role in mediating inheritance of disease risk [11]. Prenatal exposure to famine during the Dutch hunger winter of 1944 is associated with obesity with less DNA methylation of the imprinted insulin-like growth factor 2 (*IGF2*) gene in exposed offspring relative to their unexposed siblings [12, 13]. Recently, retinoid X receptor alpha (*RXRA*) promoter methylation was demonstrated to correlate with increased adiposity in two independent cohorts of children of mothers with low carbohydrate intake [14]. Increasing evidence suggests that epigenetic marks can be subjected to reprogramming in response to both stochastic and environmental stimuli, such as the in utero environment [15]. In addition, a large number of epigenetic marks are relatively stable over time [16], suggesting that those epigenetic changes acquired early in life may have a long-lasting impact on future health.

The objective of the present study was to assess the association between altered DNA methylation in neonates born with high birth weight and cardiometabolic risk parameters in preschool children, using a case–control study of preschool children born at term after noncomplicated pregnancies.

## RESULTS

### Metabolic parameters

To compare the metabolic parameters between children born with different birth weight, we measured glucose and insulin values, HOMA index, lipid profile, and uric acid in fasting conditions in children at 3–6 years of age. As shown in Table 1, when grouped by birth weight categories, the LGA children had significantly higher levels of total cholesterol (TC), low-density lipoprotein cholesterol (LDL-c), and insulin and ratio of TC/high-density lipoprotein cholesterol (HDL-c) when compared with that for the appropriate for gestational age (AGA) children; their body weight, length, and BMI were also higher. Although there was a trend to higher levels of glucose, it was not significant. Furthermore, maternal characteristics of those subjects are presented in Supplementary Table S1. Due to the matching criteria, maternal age, height, weight gain during pregnancy, and maternal occupation, education, parity, and family history showed no significance between both groups. Furthermore, there was no difference in blood pressure (BP), red blood cells, hemoglobin, platelets and serum glucose, and protein levels between mothers of AGA and LGA children. However, the weight and BMI during late pregnancy of mothers from LGA group presented significantly higher than that of mothers from the AGA group. Maternal triglyceride (TG) levels were higher in the LGA group than that in AGA group (Supplementary Table S1), whereas there was no difference in cholesterol levels.

**Table 1:** Anthropometric and metabolic parameters in children of 3–6 years grouped by birth weight

	AGA (*n*= 123)	LGA (*n*= 58)	*P* value^1^
Age (month)	55.11 ± 7.87^2^	55.02 ± 7.20	0.472
Birth weight (g)	3237.07 ± 322.88	4114.05 ± 197.45	< 0.001
Birth height (cm)	50.06 ± 1.68	50.90 ± 1.55	< 0.001
Male/female sex (n)	64/59	32/26	0.751
Weight (kg)	17.84 ± 2.75	19.75 ± 3.07	< 0.001
Height (cm)	107.59 ± 6.41	110.41 ± 4.86	0.002
BMI (kg/m^2^)	15.36 ± 1.49	16.14 ± 1.76	0.001
Weight gain (g/month)	266.45 ± 43.65	287.36 ± 62.46	0.005
Systolic BP (mmHg)	97.59 ± 10.48	98.94 ± 6.96	0.187
Diastolic BP (mmHg)	56.92 ± 8.55	58.07 ± 7.40	0.190
MAP (mmHg)	70.47 ± 8.10	71.69 ± 6.19	0.156
Pulse pressure (mmHg)	40.67 ± 9.41	40.87 ± 8.04	0.446
Serum TG (mmol/L)	0.73 ± 0.28	0.72 ± 0.28	0.487
Serum TC (mmol/L)	4.31 ± 0.74	4.62 ± 0.76	0.005
Serum HDL-c (mmol/L)	1.47 ± 0.27	1.47 ± 0.24	0.476
Serum LDL-c (mmol/L)	2.25 ± 0.54	2.46 ± 0.54	0.008
Serum TC/HDL-c	2.99 ± 0.56	3.19 ± 0.57	0.014
Fasting glucose (mmol/L)	4.78 ± 0.40	4.87 ± 0.37	0.091
Fasting insulin (uU/ml)	3.62 ± 2.04	4.19 ± 1.99	0.038
HOMA-IR	0.79 ± 0.50	0.92 ± 0.47	0.048

1 Data were analyzed by using Student’s t, and Mann-Whitney tests.

2 Mean ± SD (all such values).

3 BP, blood pressure.

4 MAP, mean arterial pressure.

### Relationship between anthropometric, BP, and metabolic parameters at 3–6 years of age

To further study the relationship between the birth weight and cardiometabolic risks, we analyzed the correlation coefficients between birth and current weight, as well as the mean weight gain, with systolic and diastolic BP and cardiometabolic parameters. Birth weight was related to cardiometabolic parameters, positively to TC, LDL-c, and the ratio of TC/HDL-c even after being adjusted by BMI and weight at 3–6 years of age (Table 2). The weight and BMI at 3–6 years old and the average weight gain were positively related to systolic and diastolic BP, fasting glucose, insulin, and HOMA index but not correlated with TC, LDL-c, and the ratio of TC/HDL-c (Supplementary Table S2). Those results suggest that excess birth weight increases the risk of cardiometabolic disease later in life.

**Table 2:** Correlation coefficients between cardiometabolic parameters with birth weight

Parameters	Birth weight			
	Non-adjusted	*P* value	Adjusted	*P* value
Systolic BP (mmHg)	0.009	0.452	0.061	0.429
Diastolic BP (mmHg)	−0.001	0.494	−0.076	0.324
MAP (mmHg)	0.003	0.484	0.081	0.293
Pulse pressure (mmHg)	0.011	0.444	0.005	0.946
Serum TG (mmol/L)	0.054	0.237	0.025	0.749
Serum TC (mmol/L)	0.255*	< 0.001	0.267*	< 0.001
Serum HDL (mmol/L)	−0.019	0.399	−0.002	0.979
Serum LDL (mmol/L)	0.294*	< 0.001	0.302*	< 0.001
Serum TC/HDL	0.217#	0.002	0.211#	0.006
Fasting glucose (mmol/L)	0.022	0.383	0.043	0.575
Fasting insulin (uU/ml)	0.047	0.264	0.105	0.173
HOMA-IR	0.043	0.284	0.103	0.181

DBP indicates diastolic blood pressure; SBP, systolic blood pressure; MAP, mean arterial pressure; HDL, high-density lipoprotein; HOMA-IR, homeostatic model assessment; and LDL, low-density lipoprotein. Adjusted correlation coefficients, after adjusted by current weight and current BMI.

* *P* < 0.001, and

# *P* < 0.01.

### DNA methylation analyses

**Figure 1:**
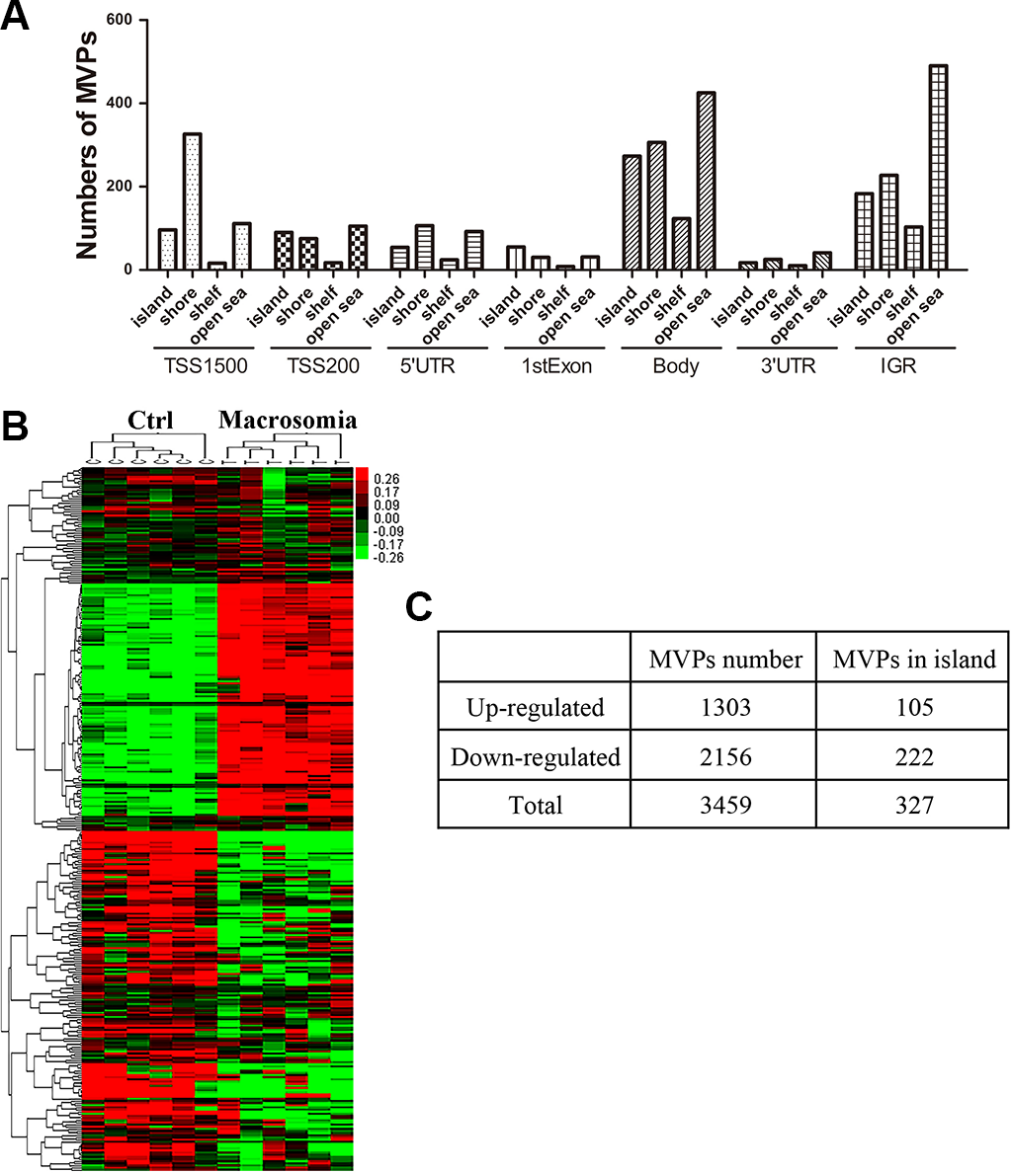
DNA methylation in genomic level altered in cord blood from macrosomia (**A**) Distribution of a total of 3459 methylation variable positions (MVPs) after the initial statistical significant filter (adjusted *P-value* < 0.05 and methylation differences of ≥ 5%) according to epigenetic/genomic feature. *Y*-axis denotes specific numbers of MVPs involved in each epigenetic/genomic feature; *X*-axis denotes genomic features (annotated as TSS1500, TSS200, 1stExon, 5´UTR, 3′UTR, gene body or IGR, andepigenetic feature-distances from a CG enriched region (CGI) (island, shore, shelf, open sea). (Abbreviations: TSS1500, within 1.5 kB of transcriptional start site; TSS200, within 200 bp of transcriptional start site; IGR, intergenic region). (**B**) Heat map including the top statistically significantly MVPs in island (*n* = 327, adjusted *P-value* < 0.05 and methylation differences of ≥ 7%). (**C**) The number of up-regulated and down-regulated MVPs with different filters.

To explore the mechanisms involved in birth weight and the risk of cardiometabolic disease in later life, we use the Illumina Human Methylation 450 BeadChip to assay genome-wide DNA methylation in umbilical cord blood from six controls and six macrosomia cases. Detailed information for the 6 pairs of selected subjects was showed in Supplementary Table S3. Results indicated that there was comparable between the two groups. Totally, 444,152 CpG loci remained for analysis after all quality control steps. We set the threshold of significance for methylation variable positions (MVPs) using adjusted *P value* = 0.05 and delta beta = 5%. Samples were successfully differentiated into control and macrosomia groups after principal component analysis. In total, there were 3459 MVPs remaining after the initial filter, and 62.3% (2156/3459) of them were less methylated. According to their different genomic features and relation to the nearest CpG islands, MVPs could be annotated as transcription start sites (TSS) 1500, TSS200, 1stExon, 5´untranslated regions (UTR), 3´UTR, gene body or intergenic region (IGR), and island, shore, shelf, or open sea. Excluding 29.0% (1003/3459) MVPs that were located on an IGR, 23.8% (585/2456), the remaining MVPs were distributed on islands (Figure 1A). Further analysis was performed in 327 MVPs (filtered by adjusted *p value* < 0.05, delta beta ≥ 7%) and CpGs were located in islands, which mapped to 213 genes. Differential DNA methylation between controls and the macrosomia group could be observed in the heatmap (Figure 1B and 1C). DNA methylation level was down-regulated in 67.9% (222/327) of MVPs. To detect the biological functions and major pathways related to these 213 genes, we performed functional enrichment analysis using Gene Ontology (GO) Tool from DAVID Bioinformatics Resources. According to the results of GO enrichment in the biologic process (Supplementary Table S4), 4 of 5 terms were related to embryo organ or system development, and the top one was “embryonic organ development” (9 genes, *P* = 5.52E- 04). “Endocytosis” (9 genes, *P* = 0.01) was found to be the most significantly Kyoto Encyclopedia of Genes and Genomes (KEGG) term (Supplementary Table S5).

### Ingenuity pathway analysis (IPA) analysis

**Figure 2:**
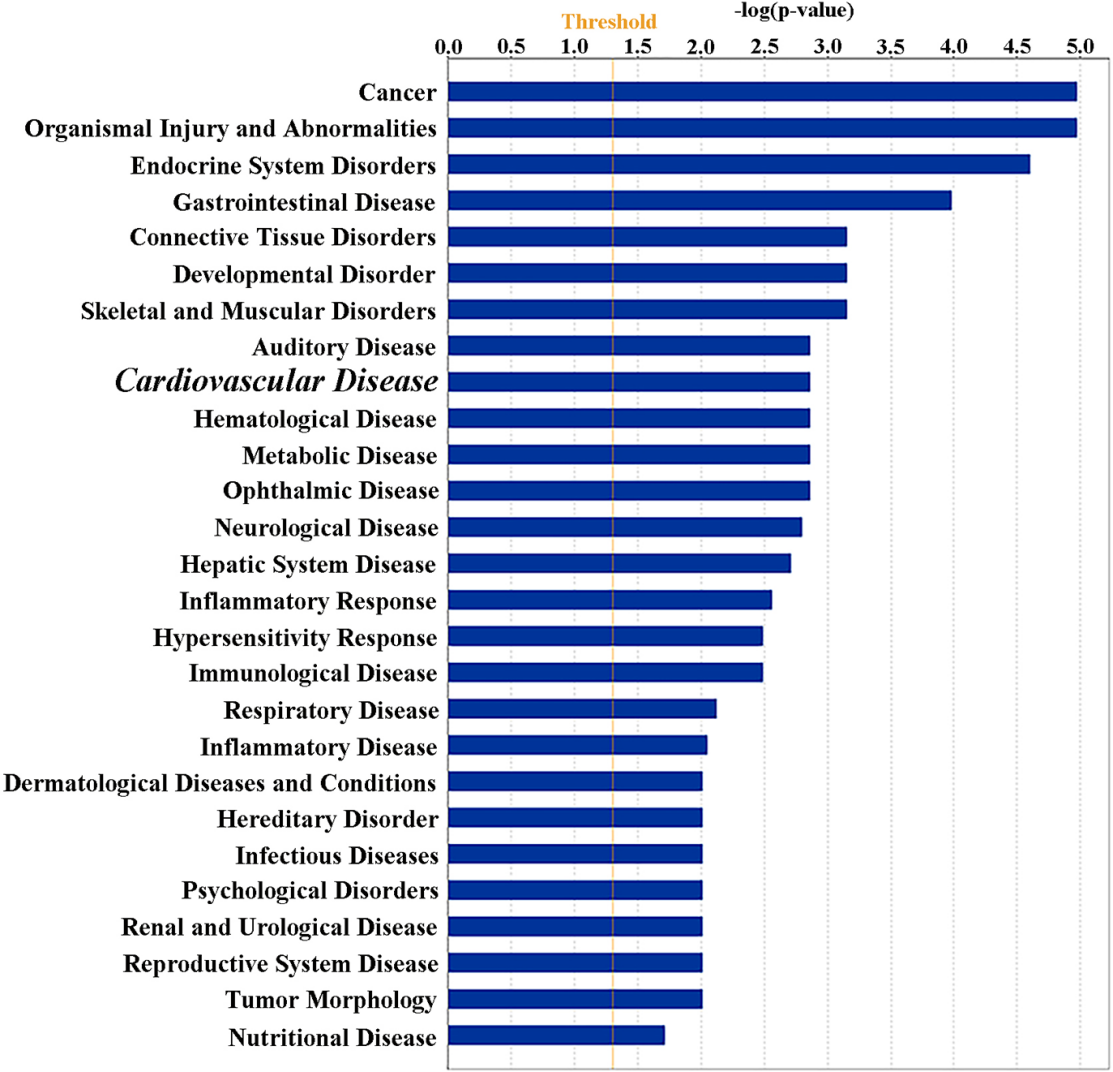
Ingenuity pathway analysis Functional classification of 213 genes mapped by the top statistically significant 327 MVPs identified between controls and the macrosomia group using the Ingenuity Pathway Analysis. “Diseases and disorders” enriched 27 terms, and including “Cardiovascular Disease” (Bigger Italic fonts).

**Figure 3:**
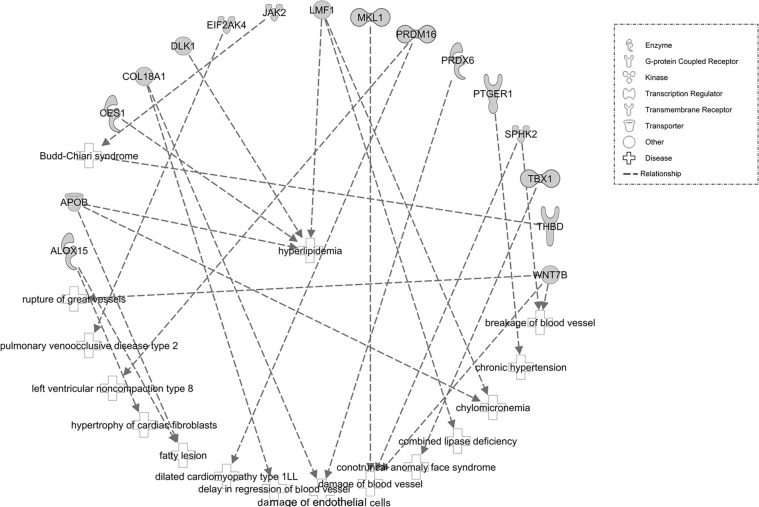
Downstream effect analysis of specific genes with differentially methylated CpGs associated with CVD For this cardiovascular function network, genes or gene products are represented as nodes, and the biological relationship between two nodes is represented as an edge. All edges are supported by at least one publication in the Ingenuity Knowledge database. The legend of the interaction network and the relationships between molecules are summarized on the right of the figure.

We next used the IPA software (QIAGEN, Redwood City, CA, USA) for functional analyses for the 213 genes. We observed 27 enriched “Diseases and Disorders” terms (Figure 2). The top items identified were: (1) “Cancer”, (2) “Organismal Injury and Abnormalities”, and (3) “Endocrine System Disorders”. In addition, “Cardiovascular Disease” (16 genes, 0.001< *P* < 0.019) was also included within the enrichment. Among 16 genes in “Cardiovascular Disease”, many genes participated in lipid metabolism (Figure 3). Four representational genes, including Apolipoprotein B gene (*APOB*), Carboxylesterase 1 gene (*CES1*), Delta Like Non-Canonical Notch Ligand 1 gene (*DLK1*), Lipase Maturation Factor 1, (*LMF1*), were contributed to hyperlipidemia. An average 10.1% lower methylation level was identified at *APOB* DMR in Chr2: 21266944–21266969, version 2009 (GRCh37/hg19), which also was annotated asTSS200-island; 21.2% lower methylation level in Chr16: 55866890, at gene *CES1* (body-island); 9.4%, 10.1%, and 12.9% varied methylation level in Chr14: 101192852, 101192860, and 101192913, respectively, annotated as gene *DLK1* (TSS1500-island); 15.8%, 15.3%, 20.2%, and 23.0% varied methylation level in Chr16: 979488, 979553, 979662, and 979898, respectively, annotated as gene *LMF1* (body-island). Arachidonate 15-Lipoxygenase gene (*ALOX15*), along with *APOB*, was referred to as fatty lesion associated in the “CVD” network, and 25.1% and 18.5% varied methylation level showed in Chr17: 4544507, 4544513 (body-island).

### Verification of the target genes

**Figure 4:**
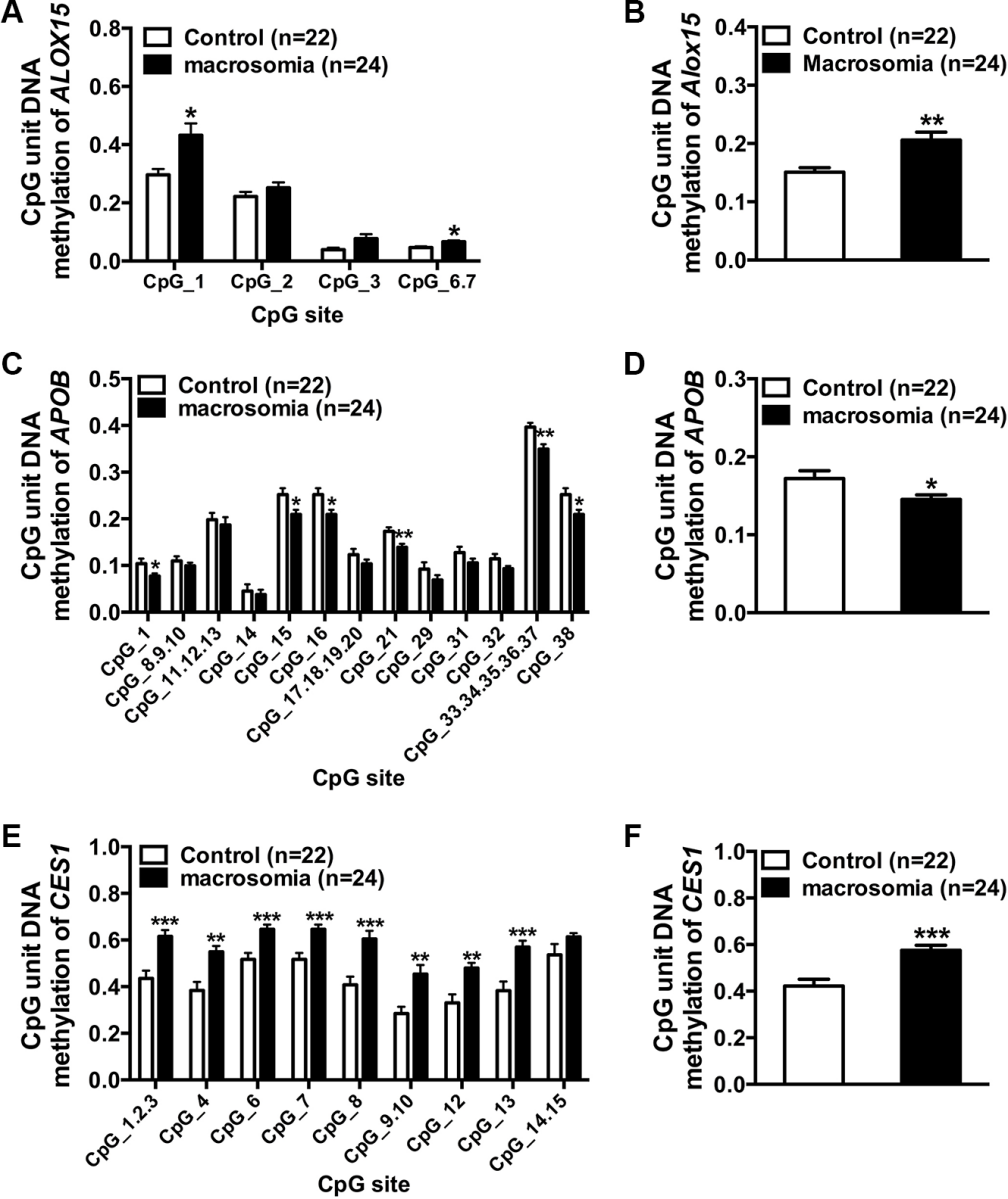
Verification of the target genes (**A** and **C** and **E**) Percentage of DNA methylation of individual CpG sites within the island at *ALOX15*, *APOB*, and *CES1* (MASSARRAY) in controls (*n* = 22) and macrosomia (*n* = 24). (**B** and **D** and **F**) Median of % DNA methylation for each region in controls (*n* = 22) and macrosomia (*n* = 24). Values (in A–F) are expressed as means ± SE, ***p < 0.0001 **p < 0.01, *p < 0.05, compared with the corresponding control.

Among those 16 genes in “CVD”, we chose the first three genes according to the alphabetical order for verification with expanded samples (control: 22, macrosomia: 24). The methylation levels of five CpG sites of *ALOX15*, 24CpG sites of *APOB*, and 13 CpG sites of *CES1* were detected (Figure 4A–E). The average methylation level of *ALOX15* in Chr17: 4544507–4544627 was significantly up-regulated in the macrosomia group (Figure 4B, P = 0.002). CpG methylation levels at sites 1, 6–7 were significantly higher in the macrosomia group (*P* < 0.01), but the methylation levels at site 2, 3 did not significantly differ (*P* > 0.05 for all) (Figure 4A). No valid data were received at site 4 and 5. The average methylation level of *APOB* in chr2: 21266623–21267021 in the macrosomia group was lower than that in the control group (Figure 4D, P = 0.02). Differential CpG methylation levels lay at sites 1, 15, 16, and 38 (*P* < 0.05) and sites 21 and 33–37 (*P* < 0.01), whereas the methylation levels of the rest of the 14 sites did not significantly differ (*P* > 0.05 for all) (Figure 4C). No valid data were received at sites 2–7, 22–28, and 30. The average methylation level of *CES1* in chr16: 55866758–55867030 was significantly increased in the macrosomia group (Figure 4F, P = 0.0006). CpG methylation levels at sites 1–3, 4, 6, 7, 8, 9–10, 12, 13, and 14–15 were significantly higher in the macrosomia group (*P* < 0.01), but the methylation levels at site 14 and 15 did not significantly differ (*P* > 0.05) (Figure 4E). No valid data were received at site 5 and 11.

## DISCUSSION

Fetal origins of cardiovascular and metabolic diseases have attracted more and more attention. Cardiometabolic risk factors, including higher BP, triglycerides, TC and LDL-c, insulin, HDL-c, and ratio of TC to HDL-c are increasingly being determined in children as well as in adulthood [17–19]. The present prospective study performed in children born at term after a noncomplicated pregnancy shows that birth weight exerted independent influences on cardiometabolic parameters at preschool age.

Barker and his colleagues were the first to find significant associations between low birth weight and the risk of chronic diseases in adulthood, including coronary artery disease, hypertension and stroke, type 2 diabetes, and osteoporosis [20]. Strong evidence from several studies indicates that individuals born with a low birth weight are more likely to present cardiometabolic complications later in life [21–24]. Recent studies in the USA, Europe, and other countries have revealed a continuous increase of mean birth weight in the past 2 decades. So far, the long-term consequences of high birth weight have not been clearly defined. A recent review examined the role of high birth weight on the development of cardiometabolic consequences (obesity, body composition, type 2 diabetes mellitus, and CVD) in childhood and adulthood [25]. Overweight and overnutrition are among the most widely recognized risk factors of metabolic diseases. The findings in a systematic review suggest that an individual with high birth weight is prone to hypertension and higher BP during childhood [26]. The subjects included in this study were only 3–6 years old, we did not find any correlation between birth weight and BP. On the other hand, it is not surprise that the average weight gain and current weight were positively correlated to BP and insulin levels. The findings indicated that “catch-up” growth correlate with some aspects of a later MetS, such as BP, which implies that a catch-up growth may be another factor linked to hypertension later in life [27]. The fetal origins of the adult disease hypothesis propose that exposures to an adverse intrauterine environment directly relates to poor nutritional status in early life and may increase their risk of adult disease, such as metabolic and CVDs [28]. Here we showed the associations of high birth weight and altered DNA methylation in neonates with cardiometabolic risk parameters in preschool children. As we all know, there were complicated maternal risks linked to birth weight [1, 10, 29]. We also investigated the maternal information during pregnancy. The results showed in Supplementary Table S1 revealed that mothers with higher BMI and serum triglyceride levels during late pregnancy are apt to born LGA neonates, which imply that prenatal exposure to an adverse intrauterine environment with potential consequences for subsequent developmental cardiometabolic diseases over the lifespan.

Accumulative epidemiological investigation has illuminated that macrosomia infants were more prone to develop CVD and MetS. However, the potential mechanisms were still unclear. Recently, epigenetics was suggested to provide a mechanistic link between environmental exposures and adult disease. DNA methylation is a well-known epigenetic modification that participates in metabolic programming during the perinatal period [30]. We have previously demonstrated in mice that the hyperglycemic intrauterine environment of GDM can increase the risk of diabetes in offspring by altering *Igf2/H19* imprinting in islets [31]. Epigenetic marks can be subjected to reprogramming in response to in utero environment, which might lead to healthy or unhealthy phenotypes, thus enabling phenotypic plasticity in the context of a fixed genotype [15, 32]. Epigenetic variation in umbilical cord blood may have a mechanistic role in metabolic disease programming through interaction of the pregnancy environment with gene function [29, 33]. Indeed, the results of DNA methylation analyses in umbilical cord blood in this study identified 327 MVPs with methylation differences of > 7% located within island, which mapped to 213 genes. Bioinformatics analysis showed many genes correlated to CVD and lipid metabolism.

The ChAMP package is a pipeline that integrates currently available 450K analysis methods. ChAMP-implemented BMIQ was identified by Marabita as an effective method [34, 35]. SNPs filter function available in this pipeline helps to prevent biases due to genetic variation in downstream statistical analyses aimed at identifying differentially methylated CpGs and focus investigation on the epigenetic factors. We also exclude probes on sex chromosomes to avoid their interference effect. However, the long period of clinical follow-up of women throughout pregnancy and their offspring in childhood was needed; it has not been possible to increase the number of samples analyzed by the 450 K plat-form in consideration of the cost of such an epigenome-wide study. It resulted in limited statistical power, weakening conclusions on methylation alteration of the individual CpG sites. Despite these limitations, verifying methylation differences on more CpG sites nearby enlarged samples and applying systematic functional analysis contributed to increasing our confidence in the results.

High birth weight contributed to epigenetic changes in embryonic organ development and morphogenesis, which probably influence an offspring’s future health. Endocytic processes, enriched in KEGG pathway analysis is a responsive mechanism to variable environments in the uterus and after birth. It (including autophagy) was linked to a wide array of vascular processes, ranging from angiogenesis to calcification of the vessel wall. Alterations in autophagic flux are also increasingly being implicated in disease processes, including atherosclerosis and pulmonary hypertension [36].

As suggested by common disease and disorder pathway analyses results, a number of the identified epivariations were correlated with CVD candidate genes. DNA methylation at gene promoter regions is often associated with transcriptional repression due to interactions between DNA methylation, methyl DNA binding proteins, and histone deacetyltransferases. In contrast, promoter hypomethylation is often associated with a euchromatic state and transcriptional permissiveness [37]. Apolipoprotein B (*APOB*) transcribes and translates into the main Apo lipoprotein of chylomicrons and LDL. LDL is considered as one of the main molecules leading to atherosclerosis and associated with cardiovascular risk [38]. Higher methylation levels in *APOB* were reported to be associated with an increased risk of having a birth weight below the 50th percentile [29]. The same relationship of methylation levels in *APOB* and birth weight was found in our study. Lower *APOB* methylation levels in specific CpGs located on islands were detected in the macrosomia group by 450K. We detected methylation of 399bp DNA surrounding the MVPs in enlarged samples by MASSARRAY and identified the same methylation differences. Delta-like homolog 1 (DLK1), an imprinted gene, is subject to multiple levels of epigenetic dosage control beyond conventional mechanisms of tissue- and temporal specific regulation [39]. Upregulation of DLK1 impairs angiogenesis by inhibiting Endothelial cell (EC) proliferation [40] and impedes the regenerative response of ECs to the proapoptotic and antiproliferative effects of oxidized LDL [41]. CES1, carboxylesterase 1, encodes a member of the carboxylesterase large family and participates in fatty acyl and cholesterol ester metabolism. An *in vitro* study showed that overexpression of CES1 in THP-1 macrophages markedly increases the rate of cholesterol efflux. Overexpression of human CES1 in macrophages increases the recruiting rates of macrophage and reduces atherosclerosis in Western diet-fed Ldlr−/−mice [42]. Differential methylation in the island and surround of CES1 was quite probably resulted in expression change [43].

In conclusion, our results suggest that high maternal TG level will dysregulate the fetal epigenome and mediate the increase of cardiometabolic disease risk in later life. Neonate born LGA presented high maternal BMI and TG levels during pregnancy. Individuals with high birth weight were accompanied by a specific pattern change of DNA methylation, including CVD candidate genes. Our data therefore provide supportive evidence that DNA methylation is involved in fetal cardiometabolic programming. The candidate genes that we have identified in this study might be severs as potential biomarkers to assess the risk of cardiometabolic disease. Therefore, our findings in this study not only extend our knowledge of pathomechanism of cardiometabolic diseases but also hold great promise for future clinical applications.

## MATERIALS AND METHODS

### Study population

Preschool children who were born at term (gestational age ≥ 37 and < 42 weeks) after uncomplicated pregnancies and in the absence of perinatal illness were invited to participate in the study between January and December 2012 from the Child Care Center of Women’s Hospital, School of Medicine, Zhejiang University. Exclusion criteria were multiple gestations, congenital anomalies, preterm infants, and small-for-gestational age. Subjects were divided according to birth weight: AGA, between 10th and 90th percentile, and LGA, > 90th percentile [44]. Fifty-eight children aged 3–6 years born LGA were enrolled according the criteria; 123 subjects born AGA were matched approximately at a ratio of 1:2 to the LGA group according to maternal age (± 1 year) and maternal weight gain (± 1 kg) during pregnancy. The study was approved by the Research and Ethics Committee of the Zhejiang Women’s Hospital, School of Medicine, Zhejiang University, Hangzhou, China, and was registered in the Chinese Clinical Trial Registry (ChicCTR-OCH-14004536, www.medresman.org). Informed consent from the parents of each participant was obtained.

The umbilical cord blood of neonates born in the Women’s Hospital, School of Medicine, Zhejiang University, was routinely collected and preserved in the biomedical sample center. The corresponding maternal and birth information was investigated. Among the enrolled subjects, 12 umbilical cord blood samples were collected at delivery, randomly involving six normal birth weight (AGA) and six macrosomia newborns, from the sample center for the DNA methylation analysis.

### Serum biochemistry parameters analysis

Serum samples were obtained under fasting conditions in the early morning and were performed for the concentration metabolic assessment. The assays for serum lipids and peripheral glucose were assayed by the clinical chemistry laboratory at the Women’s Hospital, School of Medicine, Zhejiang University (Abbott C1600, Chicago, IL, USA). The fasting insulin was tested at the same time using a chemiluminescence immunoassay (Access 2; Beckman Coulter, Fullerton, CA, USA). The homeostatic model assessment (HOMA) IR was calculated by dividing the product of insulin (microunits per milliliter) and glucose (millimoles per liter) by 22.5 [45].

### DNA preparation

Directly after delivery, umbilical cord blood was collected and stored at –80°C. DNA was extracted from buffy coats using the QIAamp DNA Mini Kit (Qiagen, Valencia, CA, USA), according to manufacturer’s protocol. Following isolation, all samples were checked for DNA quality and quantity. Those with good quality (260/280 ratio exceeding 1.8) and DNA concentration ≥ 50 ng/μl were considered to be qualified.

### 450 K BeadChip DNA methylation analysis

DNA methylation was measured using the IlluminaInfinium HumanMethylation450 BeadChips (Illumina, San Diego, CA, USA). For each sample, 500 ng of DNA was bisulfite converted using the EZ DNA Methylation Kit (Qiagen) and analyzed on HM450 Bead Chips, both according to the manufacturers’ instructions. The Illumina Genome Studio program was used for normalization and extraction of the methylated and unmethylated signal intensities. Briefly, a probe specific to each “allele” (methylated vs. unmethylated cytosines) was designed. Then, a single base extension of the probes incorporated a labeled ddNTP. Each probe signal was then used to compute a β value (β), which was a quantitative measure of DNA methylation ranging from 0 (no cytosine methylation) to 1 (complete cytosine methylation). Concretely, β was calculated as: β = intensity of the methylated allele (M) / (intensity of the unmethylated allele (U) + intensity of the methylated allele (M) + 100) [46]. Quality controls were conducted according to the manufacturer’s recommendations. Steps of DNP and Biotin staining, bisulfite conversion, extension, hybridization, target removal, and negative and non-polymorphic controls were included.

### Chip analysis methylation pipeline (ChAMP)

Analysis of 450K was performed according to the ChAMP package [47]. In brief, after raw data was loaded, three quality control images were provided. First, ChAMP filtered the data for detection (*p* < 0.01). Then, probes with a bead count < 3 in at least 5% of samples per probe were filtered out (*n* = 3324). An additional probe on the panel that contained single nucleotide polymorphisms (SNPs) or repetitive elements was removed to avoid their interference effect on measurement of DNA methylation (*n* = 37730). To adjust for type-2 bias, data were normalized with Beta-mixture quantile normalization (BMIQ). Meanwhile, an adjusted *p-value* was calculated for differential methylation using a linear model. Methylation-variable positions (MVPs) were identified for appropriate contrasts and target genes, for which MVP mapping served for bioinformatics analysis.

### DNA methylation validation

Differential methylation of Cytosine-phosphate-guanines (CpG) was validated using MassARRAYEpiTYPER assays (Sequenom, San Diego, CA, USA). We designed three primer sets by EpiDesigner software (http://epidesigner.com) to cover MVPs of gene *ALOX15*, *APOB*, and *CES1* (Supplementary Table S6). In total, 6 CpGs of *ALOX15*, 38 CpGs of *APOB*, and 13 CpGs of *CES1* were included in the target products, respectively. Each reverse primer was designed to contain a T7 promoter tag for *in vitro* transcription, and each forward primer incorporated a 10-mer tag to adjust for melting temperature differences. According to the manufacturer’s standard protocol (Sequenom), bisulfite-converted genomic DNA was prepared for polymerase chain reaction (PCR). Amplification parameters were set as follows: 95°C for 5 min, 94°C for 20 sec, 60°C for 25 sec, and 72°C for 1 min for a total of 40 cycles, with a final incubation at 72°C for 5 min. PCR products were used in *in vitro* transcription reactions (T-cleavage assay). Samples were then spotted on a 384-SpectroCHIP (Sequenom) followed by spectral acquisition on a MassARRAY analyzer compact MALDI-TOF-MS (Sequenom). Methylation data of individual units (one to three CpG sites per unit) was generated by the Epitope software (Sequenom). The non-applicable reading and its corresponding site were eliminated in calculations.

### Statistical analyses

The clinical data were normally distributed and expressed as mean ± standard deviation (SD). The independent-samples *t-test*, non-parametric test, and chi-square tests were used to evaluate the statistical significance between the two groups. Associations between parameters were assessed using the Pearson correlation coefficient. Partial correlation was used to control confounding variables. Multiple linear regression analysis, using BP values and metabolic parameters as dependent variables and birth weight, current weight, average weight gain as independent variables, was calculated. Statistical analyses were performed using SPSS version 19.0 for Windows. *P* values < 0.05 were considered statistically significant with a statistical power of 80%.

## SUPPLEMENTARY FIGURES AND TABLES




