# Supplementary material for: Altered DNA methylation in neonates born large-for-gestational-age is associated with cardiometabolic risk in children
Source: Oncotarget. 2016 Nov 18;7(52):86511–21. doi: 10.18632/oncotarget.13442 (PMC5349931; doi:10.18632/oncotarget.13442)
Supplement: Supplementary file 1 [file oncotarget-07-86511-s001.pdf]

Altered DNA methylation in neonates born large-for-gestational-age is associated with cardiometabolic risk in children

Supplementary Materials

Supplementary Table S1: Maternal characteristics in AGA and LGA groups. See Supplementary_Table_S1.

Supplementary Table S2: Correlation coefficients between cardiometabolic parameters with birth and current weight and average weight gain

Parameters	Birth weight	Average weight gain	Current Weight	Current BMI
Systolic BP (mmHg)	0.009	0.148†	0.228#	0.078
Diastolic BP (mmHg)	−0.001	0.176#	0.206#	0.143†
MAP (mmHg)	0.003	0.189#	0.244*	0.136†
Pulse pressure (mmHg)	0.011	−0.004	0.053	0.048
Serum TG (mmol/L)	0.054	0.022	0.055	0.092
Serum TC (mmol/L)	0.255*	0.015	0.023	0.029
Serum HDL (mmol/L)	−0.019	0.021	0.053	−0.056
Serum LDL (mmol/L)	0.294*	0.016	−0.006	0.044
Serum TC/HDL	0.217#	0.015	−0.039	0.088
Fasting glucose (mmol/L)	0.022	0.213#	0.214#	0.205#
Fasting insulin (uU/ml)	0.047	0.398*	0.422*	0.399*
HOMA-IR	0.043	0.385*	0.403*	0.394*

DBP indicates diastolic blood pressure; SBP, systolic blood pressure; MAP, mean arterial pressure; HDL, high-density lipoprotein; HOMA, homeostatic model assessment; and LDL, low-density lipoprotein.
*$P < 0.001$, #$P < 0.01$, and †$P < 0.05$.

Supplementary Table S3: Maternal and newborn characteristics of selected subjects

Variables	Controls (*n* =6)		macrosomia (*n* = 6)		*P* value
	Mean ± SD	Range	Mean ± SD	Range	
First trimester					
Maternal age (years)	30.0 ± 1.41	28–32	30.5 ± 0.96	29–32	0.50
Primiparous (*P* = 0)/ Multiparous (*P* =1)	2/4		3/3		0.60
Height (cm)	162.08 ± 1.56	160–164.5	162.33 ± 1.37	161–165	0.78
BMI (kg/m2)	21.55 ± 1.07	20.06–22.86	21.44 ± 0.65	20.60–22.38	0.84
Fasting glycemia (mmol/L)	4.4 ± 0.21	4.2–4.7	4.42 ± 0.24	4.1–4.8	0.91
Fasting insulin (mU/L)	6.75 ± 2.1	4.2–9.3	6.88 ± 1.81	4.7–9.8	0.91
Third trimester					
Total gestational weight gain (kg)	13.65 ± 1.91	12.0–15.8	16.78 ± 2.43	13.2–20.1	0.04
Newborn					
Gestational age at birth (weeks)	39.43 ± 0.45	39.0–40.2	39.55 ± 0.40	39.0-40.3	0.66
Male/Female	3/3		3/3		1.00
Birth weight (kg)	3295.8 ± 120.7	3150.0–3480.0	4366.7 ± 111.1	4200.0–4500.0	< 0.001

Supplementary Table S4: GO analysis

GO Term	Count	%	*P*-value
GO:0048568~embryonic organ development	9	4.25	< 0.001
GO:0048562~embryonic organ morphogenesis	8	3.77	< 0.001
GO:0048706~embryonic skeletal system development	6	2.83	0.001
GO:0045941~positive regulation of transcription	15	7.08	0.003
GO:0048704~embryonic skeletal system morphogenesis	5	2.36	0.003

Supplementary Table S5: KEGG pathway analysis

KEGG Pathway Term	Count	%	*P*-value
hsa04144: Endocytosis	7	3.3	0.01
hsa00250: Alanine, aspartate and glutamate metabolism	3	1.42	0.04
hsa04940: Type I diabetes mellitus	3	1.42	0.07
hsa04310: Wnt signaling pathway	5	2.36	0.07

Supplementary Table S6: Massarray primer sequence

Gene	Massarray primer	Number of CpGs
ALOX15	F: aggaagagagTTAGGTGATAAAGTTTGAAAGGTAGAGA<br>R: cagtaatacgactcactatagggagaaggctTCCCTTATCTAAAAA<br>-CTCAACAATCA	7
APOB	F: aggaagagagTGGATTTTGTGGTTGTTTTTTTT<br>R: cagtaatacgactcactatagggagaaggctCCCTTTAAACCTTTT<br>-ACAATCCTAAC	38
CES1	F: aggaagagagGGGTTTAGTTGTTTTAAGTTTAAGTTTT<br>R:cagtaatacgactcactatagggagaaggctCAACACAATCCCTCT-<br>AAACTACACA	15
