# Supplementary material for: Altered DNA methylation in neonates born large-for-gestational-age is associated with cardiometabolic risk in children
Source: Oncotarget. 2016 Nov 18;7(52):86511–21. doi: 10.18632/oncotarget.13442 (PMC5349931; doi:10.18632/oncotarget.13442)
Supplement: Supplementary file 2 [file oncotarget-07-86511-s002.docx]

**Supplemental Table 1: Maternal characteristics in AGA and LGA groups**

| **Variables** | AGA (n=123) | LGA (n=58) | OR (95% CI) | *P* value^1^ |
| --- | --- | --- | --- | --- |
| **Maternal** |  |  |  |  |
| Age (year) | 29.04±3.09^2^ | 29.43±3.45 | -- | 0.531 |
| Weight pre-delivery (kg) | 69.30±7.70 | 75.92±7.29 | -- | <0.001 |
| Height (cm) | 160.86±3.47 | 162.09±5.07 | -- | 0.119 |
| BMI at late pregnancy (kg/m^2^) | 26.72±2.60 | 29.08±2.82 | -- | <0.001 |
| Weight gain during pregnancy (kg) | 17.85±4.00 | 18.31±3.59 | -- | 0.286 |
| **Occupation** |  |  |  |  |
| Employed (%) | 91.87 | 88.93 | 0.65 (0.23-1.79) | 0.420 |
| Self-employed (%) | 8.13 | 12.07 | 1.55 (0.56-4.31) | 0.420 |
| **Education** |  |  |  |  |
| Middle school (%) | 24.39 | 17.24 | 0.65 (0.29-1.43) | 0.339 |
| Collage or above (%) | 75.61 | 82.76 | 1.55 (0.70-3.43) | 0.339 |
| **Parity** |  |  |  |  |
| 0 (%) | 85.37 | 84.48 | 0.93 (0.39-2.23) | 1.000 |
| 1 (%) | 11.38 | 13.79 | 1.25 (0.49-2.73) | 0.633 |
| ≥2 (%) | 3.25 | 1.72 | 0.52 (0.06-4.78) | 1.000 |
| **Family history** |  |  |  |  |
| Hypertension and/or diabetes | 17.07 | 17.24 | 1.01 (0.44-2.32) | 1.000 |
| **Blood pressure (mmHg)** |  |  |  |  |
| Basal SBP | 105.11±7.68 | 107.61±9.12 | -- | 0.410 |
| Basal DBP | 65.74±5.63 | 67.61±7.18 | -- | 0.230 |
| SBP at late pregnancy | 118.06±10.82 | 118.38±10.17 | -- | 0.419 |
| DBP at late pregnancy | 71.58±6.92 | 74.71±8.41 | -- | 0.408 |
| **Serum lipids** |  |  |  |  |
| Triglyceride (mM) | 3.34±1.78 | 4.13±2.11 | -- | 0.014 |
| Total cholesterol (mM) | 6.18±1.16 | 5.90±1.46 | -- | 0.123 |
| HDL cholesterol (mM) | 1.72±0.39 | 1.76±0.85 | -- | 0.351 |
| LDL cholesterol (mM) | 2.98±0.97 | 2.78±0.81 | -- | 0.117 |
| **Serum biochemical indexes** |  |  |  |  |
| Blood glucose (mM) | 4.54±1.15 | 4.59±0.88 | -- | 0.407 |
| Total protein (g/L) | 62.38±7.22 | 62.18±7.06 | -- | 0.315 |
| Serum albumin (g/L) | 36.37±3.21 | 35.39±7.81 | -- | 0.271 |
| **Other blood indexes** |  |  |  |  |
| Red blood cell (*10^9^/L) | 4.28±1.14 | 4.18±0.37 | -- | 0.141 |
| Hemoglobin (g/L) | 122.43±11.00 | 122.45±11.86 | -- | 0.498 |
| Platelet (*10^9^/L) | 238.27±76.34 | 241.50±79.43 | -- | 0.433 |

Note: 1, Data were analyzed by using Student’s t, Mann-Whitney U, and chi-square tests; 2, Mean±SD (all such values);
